# Supplementary material for: DNA aptamers for the recognition of HMGB1 from Plasmodium falciparum
Source: PLoS One. 2019 Apr 9;14(4):e0211756. doi: 10.1371/journal.pone.0211756 (PMC6456224; doi:10.1371/journal.pone.0211756)
Supplement: S1 Fig — See materials and methods for further details. (PDF) [file pone.0211756.s005.pdf]

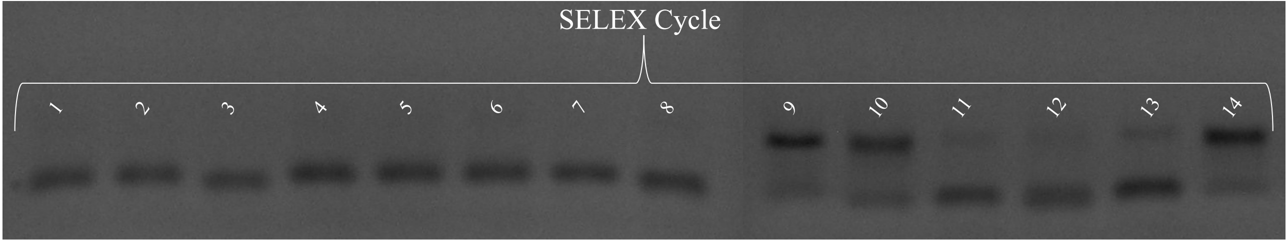

S1 Fig. Aptamer enrichment analysis by the DiVE assay. See materials and methods for further details.
