# Supplementary material for: DNA aptamers for the recognition of HMGB1 from Plasmodium falciparum
Source: PLoS One. 2019 Apr 9;14(4):e0211756. doi: 10.1371/journal.pone.0211756 (PMC6456224; doi:10.1371/journal.pone.0211756)
Supplement: S2 Fig — HMG-box540Q and PfR6FAM were used to monitor the interaction between the aptamer and the protein in real-time using a stopped-flow apparatus (Applied Photophysics, UK). HMG-box Pf Atto540Q labeled (green) or HMG-box Hs Atto540Q labeled (blue) comparison during the binding with PfR6FAM. Seven to ten replicates for each reaction were measured and averaged. Continuous lines show the fitting with exponential functions. (PDF) [file pone.0211756.s006.pdf]

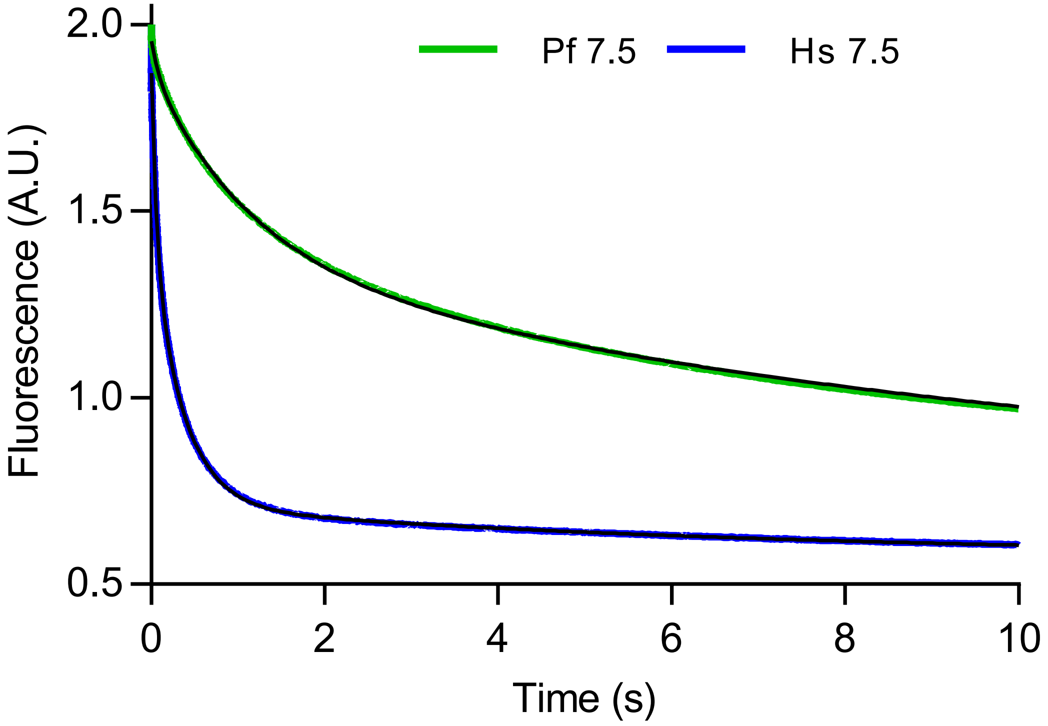

S2 Fig. Kinetics of PfR6 interaction with HMG-box *Pf* and *Hs* at pH 7.5. HMG-box^{540Q} and PfR6^{FAM} were used to monitor the interaction between the aptamer and the protein in real-time using a stopped-flow apparatus (Applied Photophysics, UK). HMG-box *Pf* Atto540Q labeled (green) or HMG-box *Hs* Atto540Q labeled (blue) comparison during the binding with PfR6^{FAM}. Seven to ten replicates for each reaction were measured and averaged. Continuous lines show the fitting with exponential functions.
