# Supplementary material for: DNA aptamers for the recognition of HMGB1 from Plasmodium falciparum
Source: PLoS One. 2019 Apr 9;14(4):e0211756. doi: 10.1371/journal.pone.0211756 (PMC6456224; doi:10.1371/journal.pone.0211756)
Supplement: S3 Fig — Here, we show the N40 variable region of the aptamers, including the flanking 5’ (TAGGGAAGAGAAGGACATATGAT) and 3’ (TTGACTAGTACATGACCACTTGA) regions. Secondary structures were predicted considering the flanking regions, at 25oC, 100 mM Na+, and 3 mM Mg2+, using the Mfold web server. (PDF) [file pone.0211756.s007.pdf]

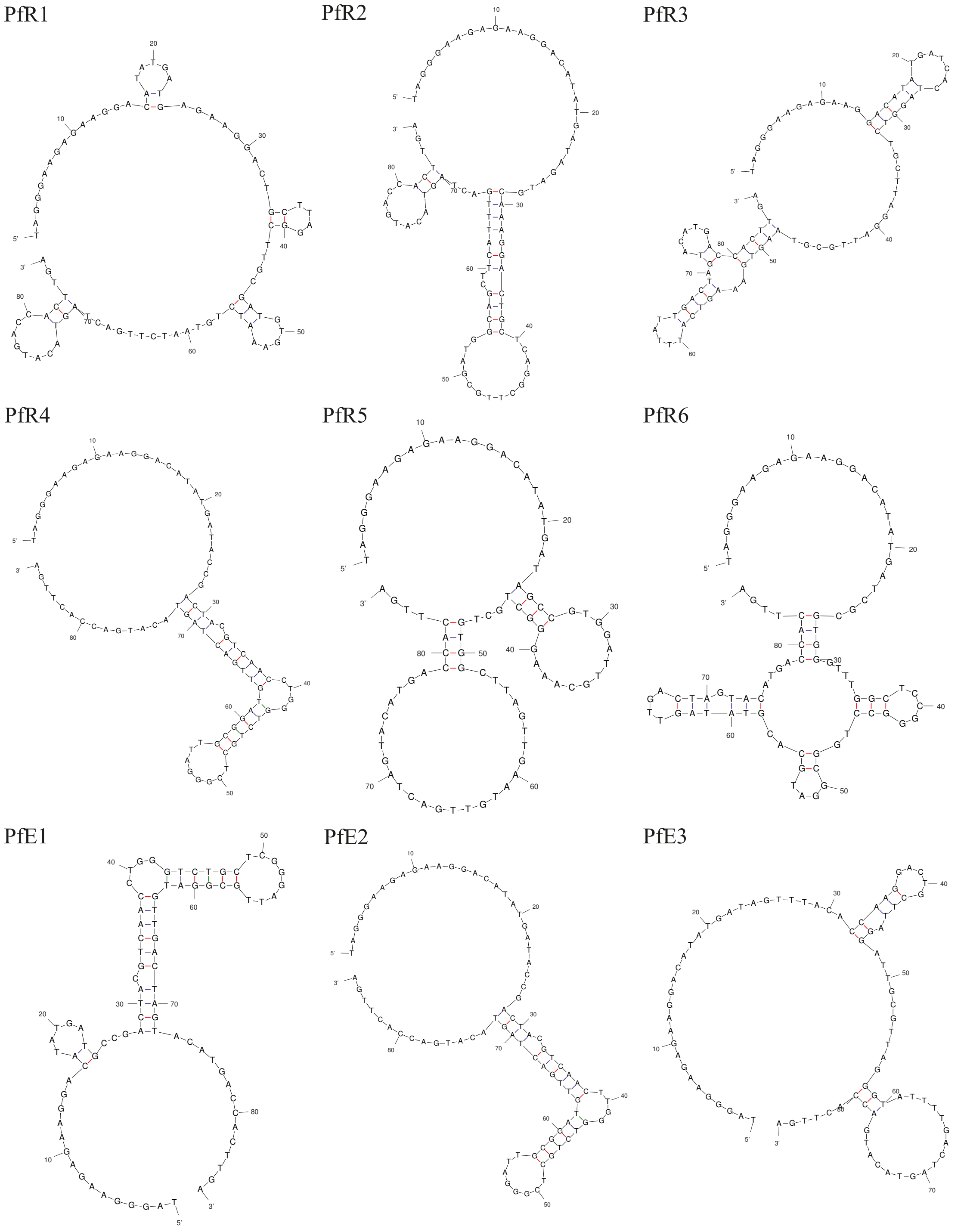

S3 Fig. Secondary structures of top-ranked and enriched aptamers. Here, we show the N40 variable region of the aptamers, including the flanking 5’ (TAGGGAAGAGAAGGACATATGAT) and 3’ (TTGACTAGTACATGACCACTTGA) regions. Secondary structures were predicted considering the flanking regions, at 25ºC, 100 mM Na^+, and 3 mM Mg^{+2}, using the Mfold web server.
