# Supplementary material for: DNA aptamers for the recognition of HMGB1 from Plasmodium falciparum
Source: PLoS One. 2019 Apr 9;14(4):e0211756. doi: 10.1371/journal.pone.0211756 (PMC6456224; doi:10.1371/journal.pone.0211756)
Supplement: S4 Fig — (A) Thermophoresis changes of PfR6 as a function of HMG-box Pf concentration. Continuous line shows the non-linear regression using a quadratic equation, allowing to estimate a Kd of 64 ± 20 nM and a thermophoretic amplitude of 7 AU. (B) As A for the PfE3 aptamer. The estimated Kd was 740 ± 120 nM and an amplitude of 16 AU. Specificity assessment for PfR6. (PDF) [file pone.0211756.s008.pdf]

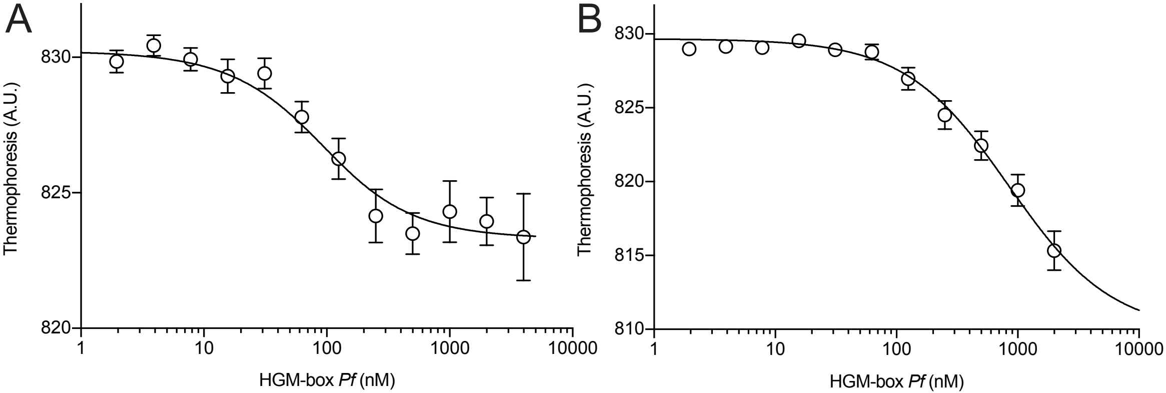

S4 Fig. MST analysis of the aptamer-HGM-box complex formation. (A) Thermophoresis changes of PfR6 as a function of HMG-box *Pf* concentration. Continuous line shows the non-linear regression using a quadratic equation, allowing to estimate a K_d of 64 ± 20 nM and a thermophoretic amplitude of 7 AU. (B) As A for the PfE3 aptamer. The estimated K_d was 740 ± 120 nM and an amplitude of 16 AU. Specificity assessment for PfR6.
