# Supplementary material for: DNA aptamers for the recognition of HMGB1 from Plasmodium falciparum
Source: PLoS One. 2019 Apr 9;14(4):e0211756. doi: 10.1371/journal.pone.0211756 (PMC6456224; doi:10.1371/journal.pone.0211756)
Supplement: S6 Fig — Binding of the selected aptamers 3D ssDNA structures to the HMG-box Pf were predicted using ZDOCK server. A) The ZDOCK score of 2,000 iterations were averaged using Graphpad Prism 7.02. B) Highest ZDOCK score of each aptamer. (PDF) [file pone.0211756.s010.pdf]

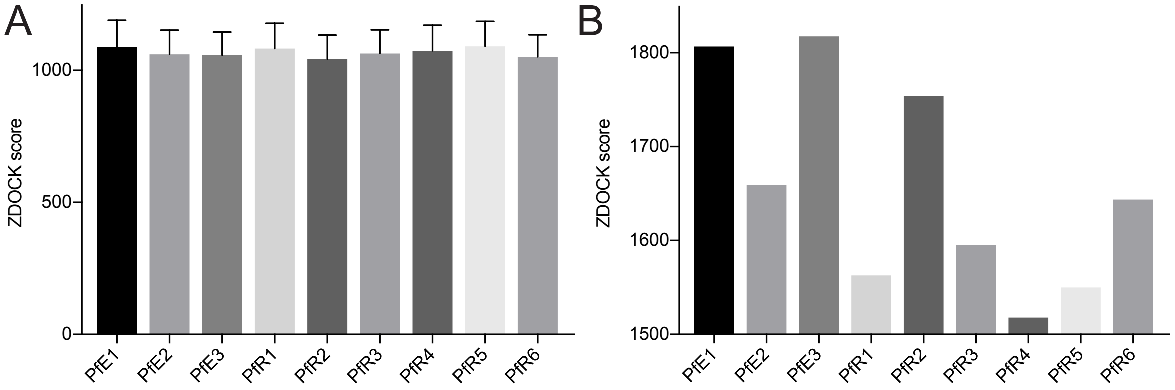

S6 Fig. Averaged ZDOCK scores for the PfR6–HMG-box *Pf* complex. Binding of the selected aptamers 3D ssDNA structures to the HMG-box *Pf* were predicted using ZDOCK server. A) The ZDOCK score of 2,000 iterations were averaged using Graphpad Prism 7.02. B) Highest ZDOCK score of each aptamer.
