# Supplementary material for: DNA aptamers for the recognition of HMGB1 from Plasmodium falciparum
Source: PLoS One. 2019 Apr 9;14(4):e0211756. doi: 10.1371/journal.pone.0211756 (PMC6456224; doi:10.1371/journal.pone.0211756)
Supplement: S1 Table — (PDF) [file pone.0211756.s011.pdf]

PlasmoDB ID	Candidate	3D Structure
PF3D7_1202900	High mobility group protein B1 (HMGB1)	1CG7 (Predicted from *S. cerevisiae*)
PF3D7_1243600	Translation initiation factor SUI1, putative	2IF1 (Predicted from *H. sapiens*)
PF3D7_0818200	14-3-3 protein (14-3-3I)	1IB1 (Predicted from *O. aries*)
PF3D7_1444800	Fructose-biphosphate aldolase (FBPA)	1A5C*
PF3D7_1365900	Tetraubiquitin	1F9J (Predicted from *H. sapiens*)

S1 Table. Potential biomarkers for *Plasmodium falciparum* infection as proposed by the algorithm in Fig 1.
