# Supplementary material for: DNA aptamers for the recognition of HMGB1 from Plasmodium falciparum
Source: PLoS One. 2019 Apr 9;14(4):e0211756. doi: 10.1371/journal.pone.0211756 (PMC6456224; doi:10.1371/journal.pone.0211756)
Supplement: S2 Table — (PDF) [file pone.0211756.s012.pdf]

SELEX	Volume (µL)	ssDNA (pmol)	ssDNA (µM)	HMG-box *Pf* (pmol)	HMG-box *Pf* (µM)	ssDNA/ HMG-box *Pf*	Incubation (min)	# washes	PCR cycles	dsDNA (pmol)	Transcription (min)	RNA (pmol)	cDNA (pmol)
1	200	1000	5.00	104.5	0.52	9.57	60	0	6	34.98	120	1008.98	168.35
2	200	150	0.75	53.2	0.27	2.82	60	1	12	43.21	120	1057.61	296.30
3	200	150	0.75	53.2	0.27	2.82	45	2	10	26.56	120	2304.53	221.47
4	200	125	0.63	53.2	0.27	2.35	30	2	10	37.41	120	857.84	142.54
5	200	125	0.63	53.2	0.27	2.35	30	3	10	28.25	120	1267.12	210.62
6	200	160	0.80	53.2	0.27	3.01	30	3	10	16.46	120	2424.24	154.51
7	200	140	0.70	53.2	0.27	2.63	30	3	8	7.11	120	1510.29	129.82
8	200	115	0.58	53.2	0.27	2.16	20	3	8	22.63	120	1231.95	51.25
9	200	50	0.25	24.7	0.12	2.02	20	3	8	41.71	120	2020.20	97.27
10	300	80	0.27	24.7	0.08	3.24	20	3	12	51.07	120	743.36	289.19
11	300	200	0.67	24.7	0.08	8.10	20	3	12	54.43	120	414.14	194.54
12	500	160	0.32	24.7	0.05	6.48	15	3	12	41.15	120	414.14	144.67
13	1000	115	0.12	15.2	0.02	7.57	15	3	22	12.35	120	1167.23	144.78
14	1500	115	0.08	15.2	0.01	7.57	15	3	15	49.20	120	2394.31	56.12

S2 Table. Reactants and products quantities and concentrations, as well as conditions in each SELEX cycle.
